# Operando Spectroscopic Analysis of Photovoltage Generation in Hematite Photoanodes

**DOI:** 10.1021/jacs.6c04274

**Published:** 2026-06-09

**Authors:** Louise I. Oldham, Daniele Benetti, Tianying Liu, Dunwei Wang, James R. Durrant

**Affiliations:** † Department of Chemistry and Centre for Processable Electronics, Molecular Sciences Research Hub, 4615 Imperial College London, London W12 0BZ, U.K.; ‡ Department of Chemistry and Schiller Institute of Integrated Sciences and Society, 6019 Boston College, Chestnut Hill, Massachusetts 02467, United States; § Department of Chemistry, 6396 University of Oxford, Oxford OX1 3TA, U.K.

## Abstract

Solar-driven water splitting requires sufficient photovoltage to drive both water oxidation and proton reduction. Understanding the factors driving and limiting photovoltage generation is therefore crucial to optimizing photoelectrode design but has proven challenging to determine under operando conditions for photoanodes driving slow multiredox reactions such as water oxidation. In this work, operando optical spectroscopy is employed to measure the hole quasi-Fermi level (*E*_F,p_) position in model hematite photoanodes as a function of applied bias and light intensity. The quasi-Fermi level splitting determined from these data are shown be in excellent agreement with the directly measured photovoltages, demonstrating the primarily electrochemical rather than primarily electrostatic origin of photovoltage in these photoelectrodes. *E*_F,p_ pinning is observed at low light intensities and biases, indicative of hole trap states lying ∼0.2 eV above the valence band edge with a density of ∼1 nm^–2^. Hole accumulation in these trap states is correlated with first order water oxidation. At higher light intensities and/or more anodic bias, E_F,p_ becomes unpinned, assigned to saturation of these trap states, and correlated with the onset of third order water oxidation to molecular oxygen. Comparison with rate law analyses for other photoanodes indicates that such hole trap states may be a ubiquitous feature of metal oxides and suggests that materials processing strategies to suppress the density of such states would be a promising strategy to enhance photoanode performance.

## Graphical abstract

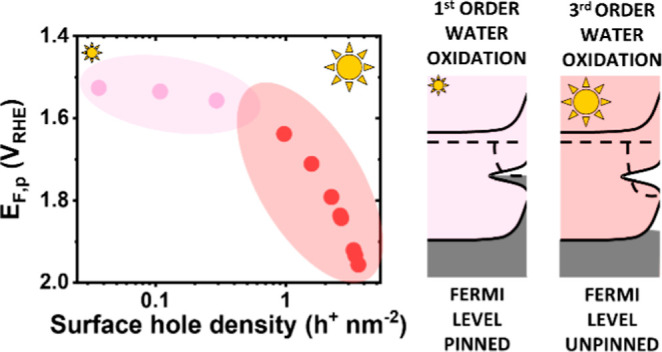

## Introduction

Solar-driven photoelectrochemical (PEC) reactions, such as water splitting and CO_2_ reduction, offer a promising pathway toward sustainable fuel production. Realizing high efficiency in these systems requires not only effective light harvesting and charge separation, but also sufficient photovoltage to drive the interfacial redox reactions.^1^ This photovoltage reduces the external voltage requirement to drive the desired reaction relative to the dark electrochemical (EC) reaction. Enhancing photovoltage generation is therefore a key goal for PEC solar energy conversion. However, in many PEC systems, operando determination of the photovoltage generated is often experimentally challenging,^2^ complicating systematic materials design strategies to enhance its magnitude.

The origin of the photovoltage in photoelectrodes has classically been interpreted as a change in electrostatic potential under illumination, driven by the generation and accumulation of photogenerated charges.^3,4^ For the classical model, which typically assumes the band edges are pinned^5^ and the surface reaction is fast,^1^ the accumulation of charge under illumination causes a reduction in band bending across the space charge layer (SCL), and thus a lowering of the electrode potential.^3,4^ Under biased, operando conditions, this results in irradiation causing a cathodic shift of the bias required for current generation – a “photovoltage”. The accumulation of charge is also often described in terms of the electrochemical potential of electrons and holes, with irradiation driving an electron and hole quasi-Fermi level splitting (QFLS).^6^ In some photoactive devices, such as silicon p–n junction solar cells or buried junction PEC systems, the change in electrostatic potential from charge accumulation has been shown to be equivalent to the change in electrochemical potential, with the photovoltage measured across the device at open circuit (*V*_OC_) being equivalent to both the change in band bending and the QFLS (Figure S1).^7,8^ However, for photoelectrodes driving slow multiredox processes such as water oxidation, the electrode energetics are no longer pinned to the electrolyte redox couple.^2,9^ In addition, for metal oxides, surface accumulation is often coupled to proton release (and vice versa for electrons),^10,11^ such that the change in electrochemical potential does not necessarily result in an equivalent change in electrostatic potential (Figure S2c).^2,12,13^ As a result, it is often not clear if photovoltage generation in metal oxide photoanodes for water oxidation is primarily electrostatic (i.e., a change in enthalpy, following classical behavior) or primarily electrochemical (i.e., a change in entropy and therefore free energy, driven by an accumulation of oxidizing species), and remains a subject of significant debate.^2,12–15^

Determination of the electrochemical versus electrostatic origins of photovoltage requires quantitative comparison of the photovoltage with the QFLS. Nevertheless, experimental determination of the QFLS in photoanodes is challenging, particularly under operando conditions. Photovoltage in photoanodes has previously been reported as the difference in open circuit potential in the light compared to the dark (Δ*V*_OCP_), analogous to the *V*_OC_ measured in photovoltaics.^1,15^ However, the bias present under operando conditions results in the extraction of electrons (for photoanodes), limiting the relevance of open circuit measurements to operando conditions. In such cases, the photovoltage can be extracted empirically from the current–voltage (JV) characteristics. It is typically defined as the difference in applied bias required to achieve the same (photo)­current density under illuminated and dark conditions^16–18^ and is sometimes referred to as the PEC voltage.^12^ An additional challenge in operando QFLS determination in photoanodes compared to photovoltaic devices is that the former features only a single electrical contact, allowing measurement of the bulk Fermi level (E_F_) position only. As such, measurements of electrode potential may not fully account for the change in electrochemical potential resulting from accumulation of valence band (VB) holes at the photoanode surface. Further considerations include whether surface states may result in Fermi level pinning, and the extent to which electron/hole accumulation at the electrode surface results in electrostatic charge accumulation or is coupled to proton uptake/release to the electrolyte and is therefore charge neutral. These considerations are particularly complex for metal oxide photoanodes for water oxidation, as investigated herein, due to the multiredox nature of water oxidation and its slow kinetics, thus requiring the accumulation of high densities of surface VB holes to drive the reaction.

Several methods have been employed to probe the QFLS of photoelectrodes directly, including a dual working electrode setup,^8,13,19–21^ vibrating Kelvin probe surface photovoltage spectroscopy^22^ and Raman scattering using a surface reporter molecule.^23^ Many such studies of QFLS in metal oxides employ single electron outer-sphere redox couples.^22,24^ Under these conditions, the hole quasi-Fermi level (*E*_F,p_) can closely track the solution redox potential (Figure S2b). In contrast, for inner-sphere, multihole reactions such as water oxidation, interfacial charge transfer is intrinsically slow. As a result, photogenerated holes accumulate at the semiconductor surface under illumination, and the position of *E*_F,p_ will shift relative to the thermodynamic water oxidation potential.^2,9,13^ Under these conditions the QFLS can be primarily governed by the energetics and population of surface-bound intermediates or catalytic states that mediate the oxidation reaction.

We have recently demonstrated that photoinduced absorption (PIA) spectroscopy combined with step potential spectroelectrochemistry (SP-SEC) enables a direct, operando measurement of the QFLS in photoanodes by quantifying photogenerated hole populations under both light and dark conditions.^25^ In the study herein, we use hematite (α-Fe_2_O_3_) as a model photoanode material to investigate how its QFLS evolves as a function of both light intensity and applied bias, and to elucidate its relationship to the photovoltage extracted from JV measurements. For the purposes of this study, we define photovoltage as the difference in applied potential required to achieve the same (photo)­current density in the light and the dark, as shown in Figure 1a. The validity of this comparison is discussed in Supporting Information. Since the light JV curve does not demonstrate an ideal fill factor, this photovoltage depends on the specific current density (or applied potential) chosen for comparison, as also illustrated in Figure 1a. It is therefore necessary to specify the bias at which the photovoltage is evaluated. For context, the *V*_OCP_ under illumination and in the dark are also shown in Figure 1a. The difference between these two values, Δ*V*_OCP_, sometimes referred to as the open circuit photovoltage, does not generally correspond to the operando photovoltage defined above, as previously shown by others,^12^ and demonstrated by us herein.

**Figure 1:**
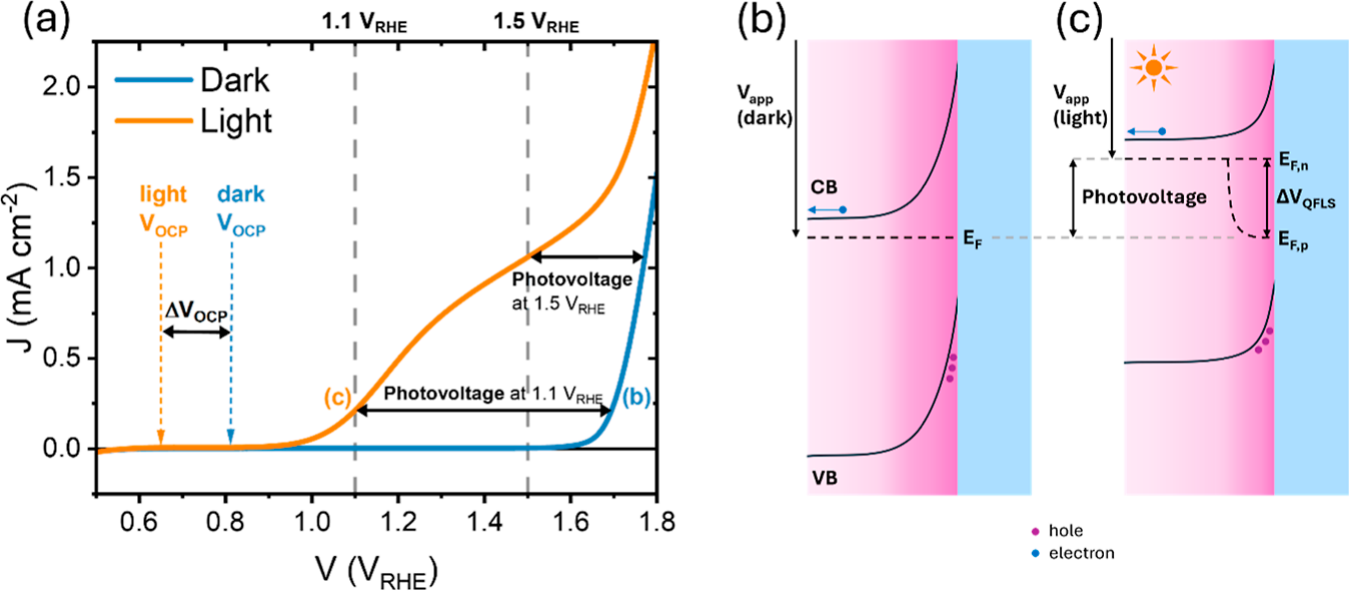
(a) Current–voltage (JV) curves of the hematite photoanodes employed in this study in the dark (blue) and under 1 sun illumination (orange). Arrows label photovoltage as the difference between the light and dark JV curves at the same current density, and Δ*V*_OCP_ as the difference between the open circuit potentials in the light and dark (photovoltage definitions discussed in the main text and in Supporting Information). (b,c) Schematic band diagrams in the dark and light, respectively, for equivalent current densities. These correlate with the positions marked (b,c) on the JV curve in (a). Δ*V*_QFLS_ = quasi-Fermi level splitting.

Several studies, including our own, have concluded that the rate of water oxidation on metal oxide photoanodes is primarily determined by the density of accumulated surface holes (or more generally “oxidizing equivalents”).^10,11,26,27^ As such, to achieve the same current density under light and dark conditions, the hole population at the surface should be equivalent, i.e. *E*_F_ (dark) = *E*_F,p_ (light),^6^ as shown in Figure 1b,c. Under illumination, Δ*V*_QFLS_ is generated in addition to the applied bias (*V*_app_(light)), allowing the same hole population, and thus the same current density, to be reached with reduced band bending compared to the dark. This situation resembles the band diagram typically drawn for open circuit conditions (Figure S2b).^1^ In Figure 1, the ΔV_QFLS_ is equivalent to *V*_app_(dark) – *V*_app_(light), i.e. the photovoltage. This band diagram is built on two key assumptions. First, we have assumed that we are operating under conditions of band edge pinning. This assumes that the applied bias drops within the SCL of the metal oxide and not across the Helmholtz layer, and that the accumulation of surface holes under irradiation does not modulate the SCL band bending (i.e., holes at the surface are proton-compensated). Second, we have assumed that the same current density in the light and the dark is correlated with the same hole population under both conditions. In this work, we test both these assumptions experimentally using operando spectroscopy and thus investigate the physical origin of QFLS at the semiconductor–electrolyte interface. By correlating spectroscopic measurements of QFLS with JV-derived photovoltage, we establish a quantitative framework for understanding photovoltage generation in our model hematite metal oxide photoanodes for water oxidation. Our spectroscopic analyses indicate pinning of the *E*_F,p_ to intrabandgap states at low bias and light intensities and unpinning at higher bias and light intensities. This transition is correlated with a transition from first to third order water oxidation reaction kinetics, thus providing a mechanistic understanding of these distinct catalytic regimes.

## Results

The hematite samples investigated in this study were synthesized by a standard hydrothermal procedure as described in previous work.^28^ The resultant films were nominally 40 nm thick with a surface roughness factor of ∼4.^29^ The JV behavior shown in Figure 1a is typical of such photoanodes.^28^ PIA and SP-SEC were used to investigate the system under PEC and EC conditions, respectively. Measurements were carried out in a three-electrode cell, using a Ag/AgCl (sat. KCl) reference electrode, Pt counter electrode and 1 M NaOH electrolyte (pH 13.4–13.7). Full experimental details can be found in the Supporting Information.

For the PIA studies, an LED light source (365 nm) of variable intensity was used to excite the sample (15 s pulse duration), and the optical and current responses were recorded simultaneously (Figure S3). The change in optical density (ΔO.D.) at 650 nm, previously assigned to photoexcited surface holes in hematite,^30–32^ was recorded as a function of time, as shown in Figure 2a. The steady state ΔO.D. values obtained can be plotted as a function of light intensity, as shown in Figure 2b. For the experimental conditions investigated in this study, the steady state current density is linear with respect to light intensity (Figure 2c). This indicates that hole accumulation does not impact the magnitude of band bending in the SCL, and thus that the system is operating under conditions of band edge pinning.^33^ This is indicative of surface hole accumulation being coupled to proton release to the electrolyte and therefore charge neutral.^10,11^

**Figure 2:**
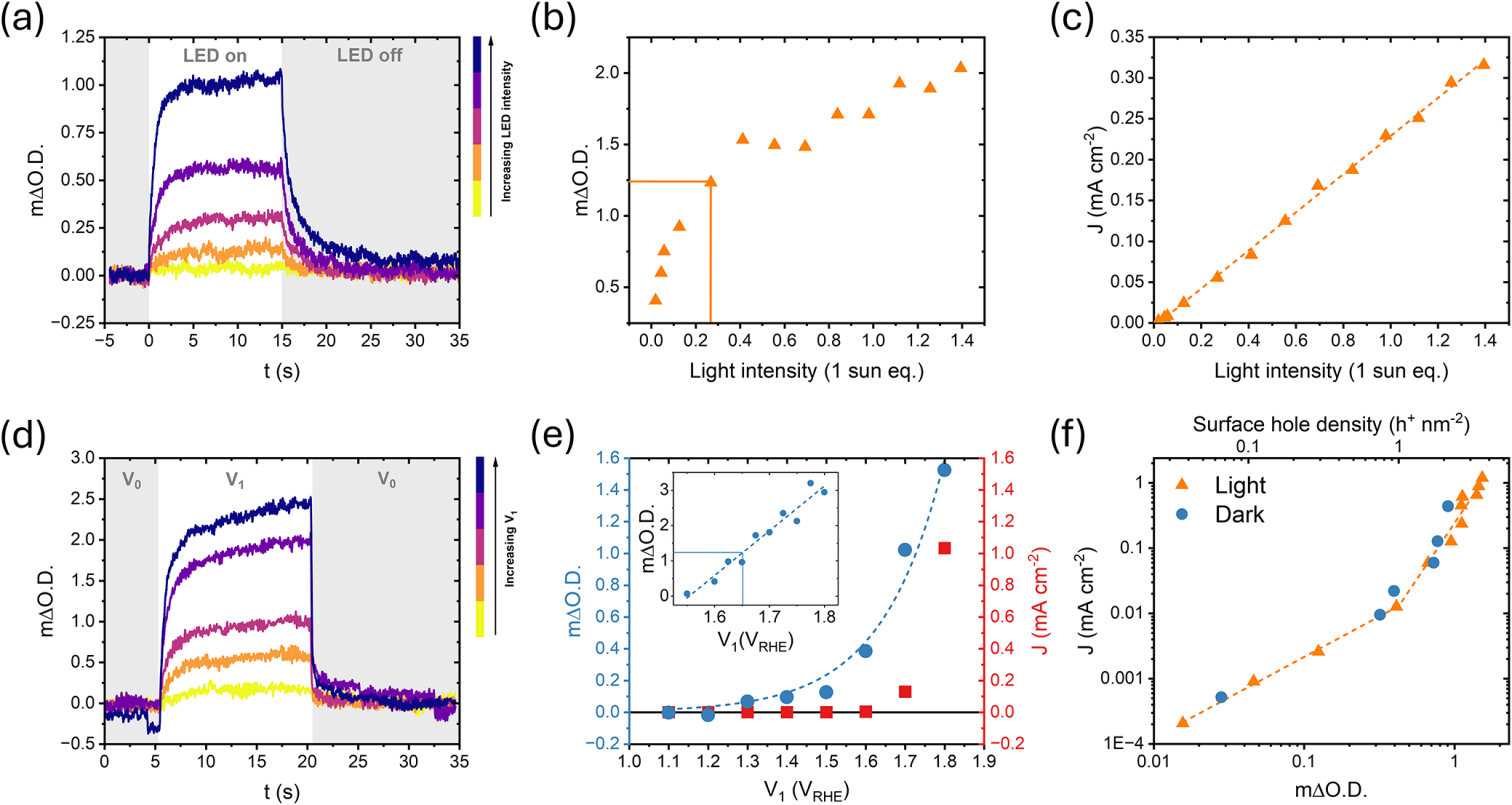
Representative PIA and SP-SEC data for hematite, used to obtain the quasi-Fermi level splitting. (a) and (d) show optical data (probed at 650 nm) obtained from PIA measurements under varying LED intensities and SP-SEC measurements under varying potential step sizes, respectively. The steady state optical signals can be plotted as a function of (b) light intensity (PIA, data shown collected at *V*_app_ = 1.3 *V*_RHE_) and (e) applied potential (SP-SEC, data in main graph collected at *V*_0_ = 1.1 *V*_RHE_, data in inset graph collected at *V*_0_ = 1.5 *V*_RHE_ on a different sample). The main figure (e) also plots the current density as a function of *V*_1_ (red squares, right *y*-axis). (c) Steady state photocurrent density, obtained during PIA measurements, plotted as a function of light intensity. (f) Rate law plot of the same hematite sample measured in the dark (SP-SEC, blue, *V*_0_ = 1.5 V_RHE_) and light (PIA, orange, *V*_app_ = 1.1 *V*_RHE_). mΔO.D. (bottom *x*-axis) converted to surface hole density (top *x*-axis) using a molar extinction coefficient of 640 M^–1^ cm^–1^ from previous work.^32^ Linear fitting of the PIA data at low surface hole densities gives a slope of 1.24 ± 0.04 and at higher surface hole densities gives a slope of 3.43 ± 0.28.

SP-SEC is the EC equivalent of PIA, exciting with a 15 s potential step rather than an LED. The potential was stepped from a fixed *V*_0_ to a variable *V*_1_, and the ΔO.D. response was recorded as shown in Figure 2d. The steady state ΔO.D. values can be extracted from this data and plotted as a function of V_1_, as shown in Figure 2e. Over a broad potential range, the dark ΔO.D. data demonstrate the expected exponential dependence on applied bias according to Fermi–Dirac statistics (see Supporting Information for further details). Within the bias window relevant to the QFLS measurements presented herein, this relationship can be accurately approximated by a linear dependence (Figures 2e inset, and S4). SP-SEC measurements were carried out at a range of *V*_0_ (Figure S5). We observed overlapping rate law behavior, independent of the starting *V*_0_. As the relationship between mΔO.D. and *V*_1_ is independent of *V*_0_ for a given sample, SP-SEC data can be collected from a single *V*_0_ value and used to compare to PIA data of the same sample at a range of applied biases under light. Control measurements discussed in Supporting Information (Figures S6 and S7) confirm that the fluorine-doped tin oxide (FTO) substrate did not contribute to the 650 nm signal and that EC water oxidation was taking place at the Fe_2_O_3_/electrolyte interface and not the FTO/electrolyte interface.

Previous PIA studies of metal oxides have observed PEC water oxidation to proceed via a population model, with a transition from first order behavior at low light intensities to third order behavior at high light intensities.^32,34,35^ More recently, this third order behavior has been observed in work from other groups.^10,26^ Our previous work on BiVO_4_ observed this same transition from first to third order behavior under EC conditions.^25^ Similar observations were made in a previous study on hematite,^36^ and were reproduced for the samples used in this study (Figures 2f, and S5a,S7,S8). These data show that the same hole population corresponds to the same (photo)­current density under both PEC and EC conditions. This overlapping rate law behavior shows that water oxidation at these metal oxide photoanodes proceeds via the same mechanism under both conditions, namely that it is driven by the accumulation of surface holes, with the key difference being in the mechanism of VB hole generation (photoexcitation for PEC conditions, Zener effect for EC conditions).^25^ To obtain the QFLS spectroscopically, the hole populations generated under both PEC and EC conditions are directly compared and must therefore participate in equivalent surface chemistry and be correlated with the same (photo)­current density. The data in Figures 2f and S7 show that these criteria are met for the hematite samples investigated in this study.

By comparison of these PIA and SP-SEC data, it is possible to find the applied potential in the dark that would be required to produce the same hole density (i.e., the same current density) under a given light intensity.^25^ For a given hole density, subtracting the applied bias used in the illuminated PIA measurement (V_app_) from the fitted dark potential (V_1_) yields the QFLS for each light intensity

1 $$\Delta V_{QFLS}=V_{1}\left(dark\right)-V_{app}\left(light\right)$$

Figure 3a shows the QFLS obtained from eq 1 using PIA and SP-SEC data (filled diamonds) as a function of light intensity at different anodic applied biases. There is a strong bias dependence of both the absolute QFLS and its light intensity dependence. At low biases, near the photocurrent onset, the QFLS is relatively large even at low light intensities but increases only weakly with illumination. In contrast, at higher biases, closer to the dark current onset, the initial QFLS is smaller but exhibits a much stronger dependence on illumination intensity. We observed similar QFLS of the same sample under both front and back illumination (Figure S9). As JV performance was higher under back illumination, we adopted this configuration for subsequent measurements. Repeat measurements across multiple samples are shown in Figure S10, demonstrating similar QFLS across samples under equivalent conditions. The experimental observations of decreasing QFLS with increasing anodic applied bias shown in Figure 3a are in agreement with previous work employing a numerical model to investigate the band diagram of a photoanode.^37^ The open circles in Figure 3a display the photovoltage extracted directly from JV curves (Figure S11). It is apparent for our samples that the JV-derived photovoltage closely matches the QFLS measured by spectroscopy at each corresponding applied bias. This both supports the validity of our QFLS measurement and indicates the photovoltage is driven by the QFLS generated under irradiation, and is therefore electrochemical in origin, as discussed further below.

**Figure 3:**
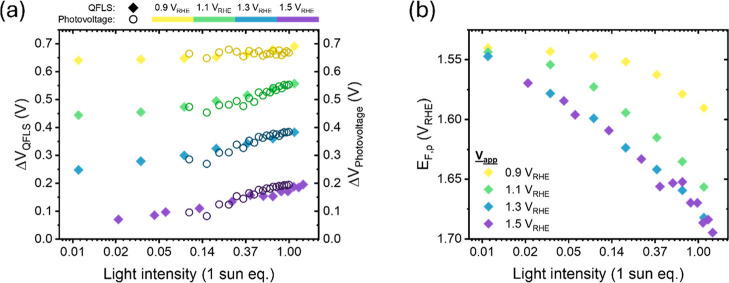
(a) Light intensity-dependence of QFLS (filled diamonds, left *y*-axis) and photovoltage (empty circles, right *y*-axis) obtained from spectroscopy and JV curves, respectively. (b) Light intensity-dependence of hole quasi-Fermi level (E_F,p_) position. Data shown for a range of anodic applied biases: 0.9 *V*_RHE_ (yellow), 1.1 *V*_RHE_ (green), 1.3 *V*_RHE_ (blue) and 1.5 *V*_RHE_ (purple). Front-side excitation in 1 M NaOH (pH 13.7). 365 nm LED used as the light source for PIA; LED solar simulator lamp used as the light source for JV curves. Light intensity is plotted on the natural log scale.

Figure 3b employs the Δ*V*_QFLS_ values determined in Figure 3a to plot the hole quasi-Fermi level, E_F,p_, position as a function of light intensity, using

2 $$E_{F,p}\left(light\right)=V_{app}\left(light\right)+\Delta V_{QFLS}$$

This equation assumes that, for an n-type semiconductor, the concentration of photogenerated electrons is negligible compared to the overall n-doping and it is possible to assume *E*_F,n_ is in equilibrium with the bulk *E*_F_,^3,6,13^ the latter of which is fixed by *V*_app_. This representation reveals that at low light intensities the *E*_F,p_ shifts to the same position (∼1.55 *V*_RHE_) independent of the applied bias. At low anodic applied bias, the *E*_F,p_ appears to be pinned at low light intensities, in agreement with previous work,^38^ and only begins to shift to more positive potentials once the light intensity exceeds ∼0.1–0.2 sun. In contrast, at higher anodic bias the *E*_F,p_ shifts progressively to more positive potentials as the light intensity increases across the full light intensity range measured, suggesting no pinning of the *E*_F,p_ under such conditions. Our observation of apparent *E*_F,p_ pinning at low anodic bias/light will be discussed further below.

In contrast to conditions of applied bias, electron accumulation is expected under open circuit conditions due to the absence of electron extraction. By recording *V*_OCP_ (measured relative to a reference electrode and therefore distinct from *V*_OC_) at each light intensity, it is possible to measure the position of the *E*_F,n_ as a function of light intensity, under the assumption that *E*_F_ = *E*_F,n_ = *V*_OCP_.^3,6,13^ PIA measurements carried out under open circuit conditions (shown in Figure S12) can be used to determine the *E*_F,p_ position by the spectroscopy method detailed above. The resulting open circuit *E*_F,p_ and *E*_F,n_ positions are plotted as a function of light intensity in Figure 4a. Note that the *E*_F,p_ and E_F,n_ positions shown in Figure 4a have been determined under the assumption that the system is still operating under conditions of band edge pinning at open circuit. This may not be the case if uncompensated charged electrons are accumulated in the SCL/at the surface at open circuit. As such, the shift in *E*_F,n_ in Figure 4a is an upper limit of this shift. As expected, given the low charge separation efficiency expected at open circuit, the *E*_F,p_ position remains largely unchanged with increasing light intensity, indicative of Fermi level pinning. The *E*_F,n_ shifts to more negative potentials with increasing light intensity, assigned to a change in electrostatic band bending as electrons accumulate in the SCL. This contrasts with the quasi-Fermi levels under applied bias, shown in Figure 4b, where the *E*_F,n_ position is fixed by the applied bias and the *E*_F,p_ position shifts more significantly with increasing light intensity. The difference between these behaviors at open circuit and under applied bias is discussed in more detail in the following section.

**Figure 4:**
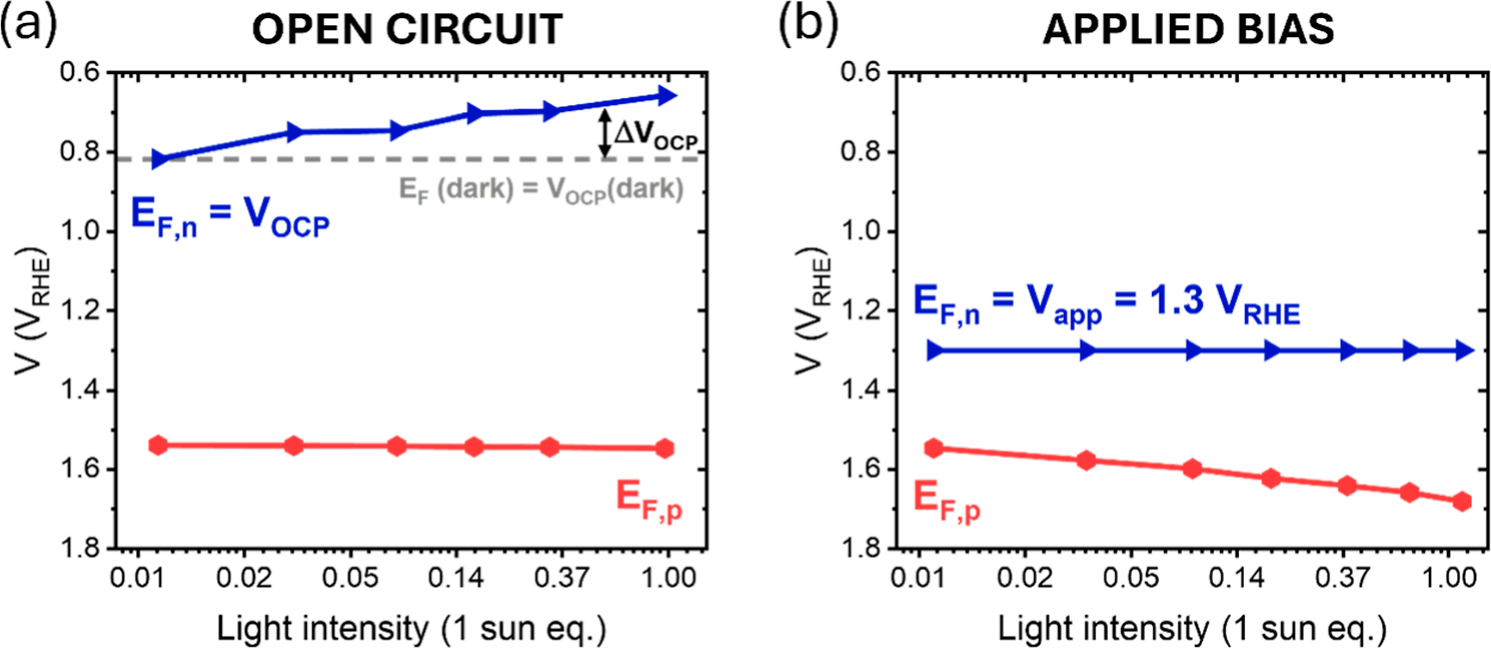
Light intensity-dependence of the electron and hole quasi-Fermi level positions (*E*_F,n_ (blue triangles) and *E*_F,p_ (red hexagons), respectively) under (a) open circuit conditions and (b) an applied bias of 1.3 *V*_RHE_. In (a), the *E*_F,n_ position was determined from measurements of the open circuit potential, *V*_OCP_. In (b), the *E*_F,n_ position is equivalent to the applied bias, *V*_app_ = 1.3 *V*_RHE_. In both plots, the *E*_F,p_ position was determined spectroscopically using PIA measurements under (a) open circuit conditions and (b) at an applied bias of 1.3 *V*_RHE_. Front-side excitation by a 365 nm LED in 1 M NaOH (pH 13.7). Light intensity is plotted on the natural log scale.

## Discussion

This study establishes an operando methodology for directly quantifying the QFLS in hematite photoanodes from minority carrier populations. The results reported herein show a quantitative agreement between the spectroscopy-derived QFLS and the JV-derived photovoltage, indicative of the QFLS driving photovoltage generation. This agreement further confirms the electrochemical, rather than purely electrostatic, origin of the operando photovoltage in hematite photoanodes. Both the absolute values and light intensity dependence of the QFLS demonstrate a strong dependence on applied bias. Furthermore, at low light intensities, the *E*_F,p_ determined from our measured QFLS is observed to pin to the same energy, independent of the applied bias. Finally, our analyses highlight the different origins and behavior of the photovoltage under operando versus open circuit conditions. These points are expanded on in the following paragraphs.

We observe a strong correlation between the JV-derived photovoltage and the spectroscopy-derived QFLS at the corresponding applied bias (Figure 3a). This provides direct experimental evidence that the internal QFLS is the origin of the JV-derived photovoltage and that, in metal oxide photoanodes under applied bias, the photovoltage is driven by the accumulation of electrochemical populations (e.g., surface VB holes for n-type materials). The equivalence of our measured QFLS and JV-derived photovoltage is evidence for the presence of band edge pinning under operando applied bias conditions. Furthermore, the observed rate law behavior, where the reaction rate depends only on the density of accumulated surface holes, implicitly requires that all surface holes are energetically equivalent, and therefore provides further evidence for band edge pinning, consistent with our observed linear dependence of photocurrent upon light intensity. The presence of band edge pinning even under conditions of surface VB hole accumulation can be attributed to surface holes corresponding to the oxidation of surface Fe–OH groups to Fe=O, with the concomitant release of a proton to the electrolyte.^10,11^ Under such conditions, the accumulation of surface holes results in no change in net surface charge and the energies of the band edges therefore remain constant relative to the redox potential of the electrolyte. This conclusion of band edge pinning is likely to be generally applicable to photoanodes exhibiting rate law behavior, which implies VB hole accumulation is charge-neutral, including BiVO_4_, WO_3_ and TiO_2_.^11,34,35^ We note that we^33^ and others^36^ have observed deviation from rate law behavior on hematite at light intensities >1 sun illumination, and have shown this deviation to be in quantitative agreement with a model of band edge unpinning.^33,39^ We have attributed this deviation to the density of surface holes exceeding the density of deprotonatable surface Fe–OH groups, resulting in net charge accumulation and likely electrostatic contributions to the photovoltage (Supporting Information).^33^ However, the study herein is limited to light intensities ≤1 sun, where rate law behavior is observed. Under these conditions, we conclude that photovoltage generation in our hematite photoanodes is not driven by a change in surface charge, and so is not purely electrostatic in origin, but rather is driven by the increase in surface VB hole population, and therefore electrochemical in origin.

It is clear from Figure 4 that *E*_F,n_ and *E*_F,p_ behave differently at open circuit compared to applied anodic bias conditions. At open circuit, photoexcited electrons cannot be extracted to the external circuit, resulting in an accumulation of uncompensated negative charge away from the semiconductor surface. This reduces the electrostatic band bending, causing a negative shift in *E*_F,n_ position with increasing light intensity. In contrast, our optical data indicate that, under these open circuit conditions, *E*_F,p_ appears to pin under irradiation at ∼1.55 *V*_RHE_ (see discussion below of the origin of this apparent pinning). It is apparent that the change in *V*_OCP_ with irradiation (Δ*V*_OCP_ = *E*_F_(dark) - *E*_F,n_(light)) is much smaller than the open circuit QFLS (Δ*V*_QFLS_ = *E*_F,p_(light) - *E*_F,n_(light)), consistent with the JV curves shown in Figure 1a. Δ*V*_OCP_ measurements only account for changes in the bulk *E*_F_ (*E*_F,n_) position and do not account for changes in the *E*_F,p_ position. This agrees with previous work which has shown that the open circuit photovoltage is not equivalent to the QFLS in the case of slow redox couples such as water oxidation.^2^ It can be concluded that the primary origin of the change in *V*_OCP_ with light irradiation on our hematite photoanodes is electrostatic in origin, driven by charge-uncompensated electron accumulation. This behavior is in clear contrast to photovoltage generation under operando anodic bias conditions, which is instead governed predominantly by the accumulation of charge-neutral surface VB holes, and is therefore electrochemical in origin. As such, it is apparent that, in contrast to previous analysis of more classical semiconductors (Si, GaAs) in nonaqueous electrolytes,^40^ measurements of open circuit potential as a function of light intensity are not a meaningful indicator of photovoltage and QFLS generation under operando, applied anodic bias conditions for water oxidation at metal oxides.

The apparent pinning of the *E*_F,p_ at 1.50–1.55 *V*_RHE_ even at the lowest light intensities, as illustrated in Figure 3b, suggests the presence of intrabandgap states above the valence band edge (VBE), which act as hole traps. Various studies have reported the presence of deep surface trap states.^26,41,42^ The chemical origin of these states remains unclear, with possible assignments including polaronic hole states or Fe vacancies.^43–47^ The exact energy of these states varies across studies, and has been reported to be up to 0.6 eV above the VBE.^46^ This may reflect differences in defect density, doping, measurement conditions or material processing. These states are distinct from those associated with oxygen vacancies, which lie close in energy to the conduction band (CB) and have been widely studied in metal oxides, including hematite.^48–50^ Regardless of their microscopic origin, our measurements establish the energetic position of these states close to the VBE and their key role in governing hole accumulation and water oxidation kinetics under operando conditions. Our observation of *E*_F,p_ pinning indicates these hole trap states must be oxidized before holes can accumulate in the VB and the *E*_F,p_ can shift closer to the VBE. It is not possible to distinguish spectroscopically between holes associated with these trap states and VB holes, as both species show a broad absorption feature in the visible region (Figure S13). The measured optical signal will therefore contain contributions from both trapped and VB holes. However, this will not affect the QFLS determined from such measurements because the QFLS depends only on the total concentration of accumulated surface holes and is independent of the chemical nature of these holes.

Strikingly, there is a strong correlation between the surface hole density at which the E_F,p_ unpins from these deep trap states, and the transition from first to third order reaction kinetics. Figure 5 shows the *E*_F,p_ position as a function of surface hole density alongside the rate law extracted from PIA measurements on the same sample. The transition from first to third order behavior occurs at ∼1 h^+^ nm^–2^, in agreement with previous work^32^ and corresponding to a surface coverage of ∼11%.^10^ As shown in Figure 5a, the E_F,p_ starts to shift closer to the VBE at surface hole densities >1 h^+^ nm^–2^. This indicates the surface density of these hole traps is of the order of 1 nm^–2^. The observation that *E*_F,p_ remains pinned in the regime of first order kinetics suggests that this first order behavior is governed by holes occupying trap states, rather than by VB holes. In contrast, once *E*_F,p_ begins to unpin, coinciding with a rise to third order kinetics, the dominant mechanism likely involves cooperative or multihole transfer from VB holes. Recent work in our group employed photoelectrochemical-mass spectrometry to directly quantify the products of water oxidation on Fe_2_O_3_ and observed high Faradaic efficiency of oxygen only after the transition from first to third order behavior.^51^ These data, in conjunction with the results of the study reported herein, provide strong evidence that the trap states observed at ∼1.50–1.55 *V*_RHE_ are not active for third order oxygen evolution reaction (OER) but instead oxidize water through a first order mechanism. The mechanisms of these two regimes have been discussed in detail in other work, where^•^OH radicals leading to H_2_O_2_ have been proposed as a likely product of first order water oxidation.^11^ We anticipate the observed pinning behavior to be dependent on electrolyte pH, which will affect the density of surface OH groups. An investigation of pH-dependence was beyond the scope of the current work.

**Figure 5:**
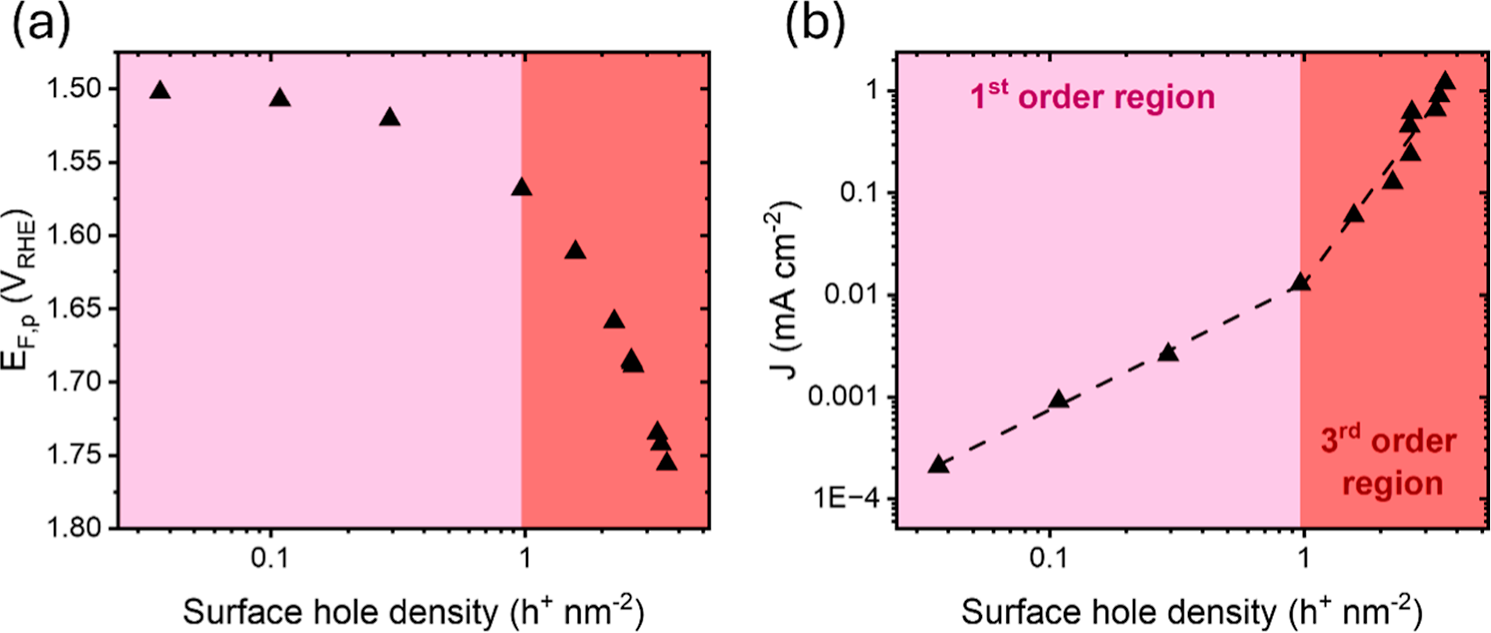
(a) Hole quasi-Fermi level (*E*_F,p_) position as a function of surface hole density and (b) rate law plot for the same hematite sample. Back-side excitation by a 365 nm LED under an applied bias of 1.1 *V*_RHE_ in 1 M NaOH (pH 13.7). First order region shaded pink, third order region shaded red.

The presence of intrabandgap states close to the VBE limits the photovoltage that can be generated under low bias/low light intensity conditions. This has been observed previously in a transient photovoltage study of hematite photoanodes, where the observed photovoltage was lower than that predicted from JV characteristics at low light and bias, and was suggested to be a result of Fermi level pinning to surface states.^38^ With the spectroscopic techniques employed herein, we are able to correlate this pinning to first order kinetics. The density of such states is therefore likely to influence the onset potential of the photoanodes, with a higher density of trap states delaying the transition to third order kinetics and anodically shifting the photocurrent onset potential.

From the observations made in this work, we can propose a band diagram of the Fe_2_O_3_/electrolyte interface under different biases and different light intensities, as shown in Figure 6. The VB position was taken from several published works and adjusted to account for pH.^52–54^ The optical bandgap obtained from UV–vis spectra of these samples was 2.1 eV (Figure S14), in agreement with literature values of 2.0–2.1 eV.^55^ Previous work has shown that, in metal oxides, polaron formation on ultrafast time scales results in a practical bandgap ∼500 meV smaller than the measured optical bandgap.^56–58^ The bulk *E*_F_ is fixed by the applied bias. As hematite is an n-type semiconductor, the population of n-type carriers generated under illumination is negligible compared to the n-doping of the semiconductor and we can therefore assume the *E*_F,n_ = *E*_F_.^3,6,13^ *E*_F,p_ positions were obtained from the spectroscopy measurements reported in this study.

**Figure 6:**
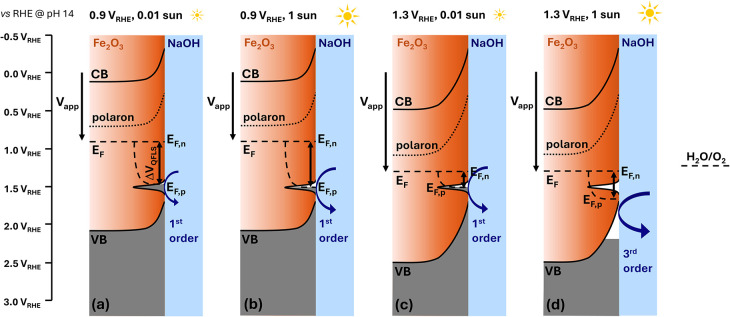
Schematic band diagrams of the hematite/electrolyte interface under illumination. Shown at an applied bias of 0.9 V_RHE_ (left panels) and 1.3 V_RHE_ (right panels) at two different light intensities. Δ*V*_QFLS_ is indicated by black double-headed arrows; hole accumulation is indicated by white shading.

Figure 6 shows that at low anodic biases close to the photocurrent onset potential, the E_F,p_ is pinned to deep states located ∼150–200 mV above the VBE. Holes occupying these states participate in first order water oxidation kinetics. As light intensity is increased, these trap states begin to fill up (i.e., become oxidized). However, strong recombination prevents sufficient hole accumulation to saturate them. As a result, the *E*_F,p_ is only able to shift closer to the VBE at sufficiently high light intensities.

At higher anodic bias, the *E*_F,p_ initially jumps to these same states at low light intensities (Figure 6c) and first order behavior is again observed. Several previous studies have shown that the increased band bending under stronger bias improves charge separation and reduces recombination losses, allowing for accumulation of larger hole populations with increasing light intensity.^50,59,60^ This is supported by normalized negative current transients, which show slower recombination at higher bias under equivalent illumination (Figure S15). As a result, once the trap states are fully saturated, additional photogenerated holes accumulate in the VB, shifting *E*_F,p_ closer to the VBE. These VB holes are catalytically active and give rise to third order water oxidation kinetics. It is likely that sample-to-sample variation in the density of deep midgap states is related to the variation in JV performance observed across samples (Figures S4b and S10c).

The model proposed in this study should be applicable to all metal oxides that exhibit rate law behavior (e.g., BiVO_4_, TiO_2_, WO_3_),^11,34,35^ and where the same hole density is correlated with the same current density under both PEC and EC conditions. Notably, in contrast to several previous studies,^22,24^ this spectroscopic method of accessing the QFLS is not limited to fast redox couples and can also be applied to slow inner sphere redox reactions such as water oxidation, as it does not require an assumption that the *E*_F,p_ is able to rapidly equilibrate with the redox potential of the electrolyte. As this model is currently limited to conditions of band-edge pinning and charge-compensated surface-hole accumulation, it would need to be modified for photoelectrodes where interfacial charge transfer is instead governed primarily by space-charge fields or buried-junction energetics, as is commonly discussed for nonoxide semiconductor/liquid junctions such as Si, GaAs, InP, and chalcogenides.^61^ Furthermore, our recent work has shown that rate law behavior in metal oxides is maintained over a finite range of light intensities and surface hole densities, beyond which a transition to Butler–Volmer-type kinetics occurs,^33^ thereby suggesting that there is a regime of validity for the present analysis (see Supporting Information for further discussion).

## Conclusion

In this work, we have experimentally demonstrated that photovoltage generation in metal oxide photoanodes driving water oxidation under operando, anodic applied bias conditions is driven by QFLS caused by the accumulation of charge neutral VB holes. As such we conclude that under these operando conditions, the origin of the photovoltage is not electrostatic, but rather electrochemical, driven by accumulation of an increasing density of enthalpically equivalent surface holes.

We have demonstrated that, in these hematite samples, the same hole population is correlated with the same current density in both the light and the dark. Furthermore, the linear dependence of current on light intensity under our experimental conditions confirms operation within the regime of band edge pinning, as also supported by our observation of rate law behavior. Taken together, these findings validate the two assumptions underlying the band diagrams presented in Figure 1 and provide a consistent physical picture of how photovoltage is generated under applied bias in such PEC systems. The model presented in this work can be applied to other metal oxide photoanodes that meet these two criteria.

The kinetic behavior observed in this work can be understood through a population model that links the hole species to the reaction order. At low surface hole densities, *E*_F,p_ is pinned to hole trap states lying 150–200 meV above the VBE, and first order water oxidation kinetics are observed. This suggests that holes trapped in these states drive this first order reaction, assigned previously to^•^OH radical formation. As the surface hole density is increased above the density of these trap states (∼1 nm^–2^), achieved by higher light intensity or higher anodic bias, E_F,p_ becomes unpinned and shifts closer to the VBE, marking the onset of third order kinetics. This transition occurs at a surface hole density of ∼ 1 h^+^ nm^–2^, consistent with previous work^32^ and supporting the idea that multihole reaction pathways are only accessible once VB holes accumulate. Comparison with analogous first/third order rate law behavior in other metal oxides (e.g., BiVO_4_) suggests the presence of such hole trap states may be a common feature of metal oxide photoanodes. Our observation of E_F,p_ pinning at low light irradiances/anodic biases reveals the importance of these hole trap states in limiting photovoltage generation and suggests that suppression of the density of these states is likely to be a promising strategy to enhance photoanode performance.

This investigation has focused on understanding the nature of photovoltage in metal oxide photoanodes under applied bias, highlighting the role of QFLS and surface carrier accumulation. When translating these insights to bias-free photocatalyst systems, such as particulate suspensions for overall water splitting, the generation of photovoltage becomes even more critical. In such systems, there is no external potential to assist charge separation or drive redox reactions; instead, the full photovoltage must arise internally from the difference between the *E*_F,n_ and *E*_F,p_. Our findings suggest that trap-state occupancy, surface energetics, and interfacial recombination all play central roles in determining the extent of this splitting. For example, our recent studies of both BiVO_4_ and Al/SrTiO_3_ photocatalyst particles for water oxidation both provided evidence for hole trap states which could be saturated under prolonged 1 sun irradiation, most likely analogous to the hole trap states indicated by our observation of *E*_F,p_ pinning herein.^62,63^ Applying the population-based framework applied herein to unbiased photocatalytic systems could help clarify how much of the absorbed photon energy is retained as usable driving force and guide the design of photocatalysts that maximize internal photovoltage for efficient, standalone solar fuel production.

## Supplementary Material



## Notes

Experimental information; supplementary band diagrams, supplementary spectroscopy and photoelectrochemical figures; UV–vis spectrum; Mott–Schottky measurements; supplementary discussions. The data needed to reproduce Figures 1–5 are available via Zenodo (PDF)
