# Supplementary material for: Operando Spectroscopic Analysis of Photovoltage Generation in Hematite Photoanodes
Source: J Am Chem Soc. 2026 Jun 9;148(24):24964–73. doi: 10.1021/jacs.6c04274 (PMC13307358; doi:10.1021/jacs.6c04274)
Supplement: Supplementary file 1 [file ja6c04274_si_001.pdf]

Operando spectroscopic analysis of photovoltage generation in hematite photoanodes

Louise I. Oldham[1], Daniele Benetti*[1], Tianying Liu[2], Dunwei Wang[2] & James R. Durrant*[1,3]

[1] Department of Chemistry and Centre for Processable Electronics, Imperial College London, London SW7 2AZ, UK.

[2] Department of Chemistry and Schiller Institute of Integrated Sciences and Society, Boston College, Chestnut Hill, Massachusetts 02467, USA.

[3] Department of Chemistry, University of Oxford, Oxford, OX1 3TA, UK.

* Corresponding authors

Daniele Benetti, d.benetti@imperial.ac.uk

James R. Durrant, j.durrant@imperial.ac.uk; james.durrant@chem.ox.ac.uk

Supporting Information

1. Experimental 2
   1.1. Fe_2O_3 film synthesis 2
   1.2. Photoelectrochemical (PEC) measurements 2
   1.3. Photoinduced absorption spectroscopy (PIA) 2
   1.4. Step potential spectroelectrochemistry (SP-SEC) 3
   1.5. UV-vis spectroscopy 3
2. Supplementary Figures 3
3. Supplementary Note 1: Comparison of PEC and EC current and hole densities 11
4. Supplementary Note 2: Ideality of system..12
5. Supplementary Note 3: Control measurements to confirm EC water oxidation occurs at the Fe_2O_3/electrolyte interface 13
6. Supplementary Note 4: Light intensities > 1 sun..14
7. References..14

1. Experimental

1.1. Fe_2O_3 film synthesis

Fe_2O_3 samples were synthesised by a standard hydrothermal procedure described in previous work, with two regrowth cycles.[1,2] The resultant films have a nominal thickness of 30-50 nm, with a surface roughness factor of ~4.[3]

1.2. Photoelectrochemical (PEC) measurements

All photoelectrochemical (PEC) measurements were carried out in a three-electrode cell setup with a Pt mesh or coiled wire counter electrode and Ag/AgCl (sat. KCl) reference electrode. Potentials were converted from $V_{Ag/AgCl}$ to V_{RHE} using the Nernst equation. The electrolyte was air-saturated 1M NaOH, pH 13.4-13.7.

Current-voltage (JV) curves were measured with a scan rate of 0.02 V s^{-1}. For PEC measurements under a 1 sun simulator, an Ossila LED array at 100 mW cm^{-2} irradiation was used as the light source and the potential was applied by an Autolab PGSTAT302N potentiostat. For PEC measurements in the spectroscopy setups, a 365 nm LED was used as the light source and the potential was applied by an Autolab PGSTAT101 potentiostat. A 365 nm LED with a light intensity of ~13 mW cm^{-2} gives the same current response in hematite as the 1 sun lamp. For all setups, the potentiostats were controlled by NOVA software.

Mott-Schottky measurements (shown in Figure S16) were carried out using an Autolab PGSTAT204 potentiostat over the non-Faradaic bias region with a modulation of 10 mV at a frequency of 1 kHz. A 365 nm LED was used as the light source.

1.3. Photoinduced absorption spectroscopy (PIA)

Photoinduced absorption spectroscopy (PIA) is a pump-probe spectroscopy technique that simultaneously detects the change in probe light absorption and current of a sample in response to an LED light pump. PIA measurements were carried out on a homebuilt system.

The probe light source was a tungsten halogen lamp (Osram 6460 HLX bulb, housed in Bentham IL1 and powered by a Bentham 610 power supply). Two monochromators (OBB), one placed before the sample and one placed after the sample, were used to select for the specific wavelength. A colour wheel (ThorLabs FW102C) was used before the second monochromator. A series of focusing and collimating lenses were used to focus the probe light on the sample and then into the Si diode detector (Hamamatsu) and photodiode amplifier modules (Costronics). The change in absorption (optical density, O.D.) was recorded by a DAQ card (NI USB-6361). The applied bias (V_{app}) was supplied by a potentiostat (Autolab PGSTAT101) which also recorded the photocurrent of the sample in response to the LED

pump. This current output was recorded by a digital phosphor oscilloscope (Tektronix DPO 3012) and converted into a current density. The potentiostat was controlled by NOVA software. The pump was a 365 nm LED (powered by an Aim-TTi QL564P power supply) and triggered electronically using a MOSFET (ZVN 430 6A). For this study, the LED was on for 15 s and off for 20 s, with 5-20 averages typically taken per measurement (note that for the PIA spectra at low light intensities in Figure S11, up to 100 averages were taken). For cases where a B test was conducted, a shutter blocked the probe light from reaching the sample and detector and these B test measurements were subtracted from the A test measurements to give the final recorded signal. Initial control measurements with a B test showed no photoluminescence of the sample so all subsequent measurements were therefore conducted with no B test.

The whole system was controlled by custom LabVIEW software, which was used to trigger the start of each measurement (i.e. DAQ card and oscilloscope to start recording and the MOSFET to turn the LED on).

1.4. Step potential spectroelectrochemistry (SP-SEC)

Step potential spectroelectrochemistry (SP-SEC) measurements were carried out on the same spectroscopy setup as PIA but used a stepped potential to pump the system rather than an LED. The potentiostat, controlled by NOVA, was used to apply a square wave potential to the sample. The system was held at V_0 for 5 s, held at V_1 for 15 s and then returned to V_0 again for 15 s. The DAQ card and oscilloscope were triggered to start recording using a trigger cable directly from the potentiostat. The optical signal and current response were recorded in the same way as for PIA.

1.5. UV-vis spectroscopy

UV-vis measurements were carried out on an Agilent Cary 60 spectrophotometer. Blank FTO was used for the baseline measurement. The sample was then scanned with baseline correction from 800-200 nm with 1 nm intervals at a scan rate of 600 nm min^{-1} . The obtained spectrum, shown in Figure S14, shows a band edge absorption ~590 nm, corresponding to an optical bandgap of ~2.1 eV.

2. Supplementary Figures

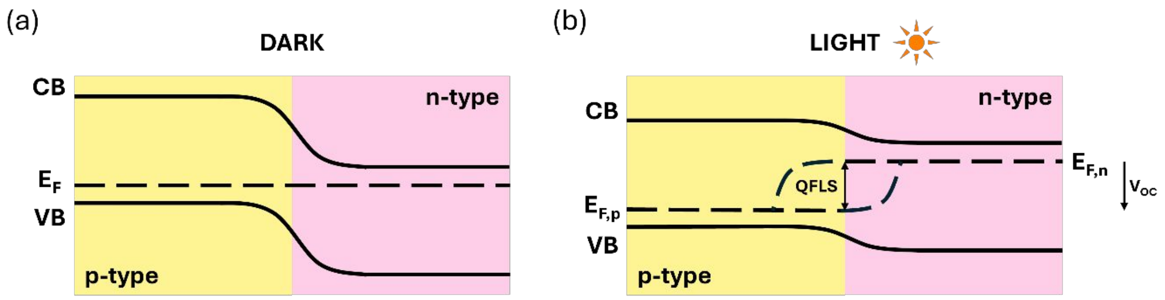

Figure S1. A schematic diagram of the band bending at the interface of a p-n junction (a) in the dark and (b) under illumination. In the light, quasi-Fermi level splitting (QFLS) at the interface of the two semiconductors separates the hole quasi-Fermi level ($E_{F,p}$) and the electron quasi-Fermi level ($E_{F,n}$) and is equivalent to the open circuit voltage (V_{OC}) measured across the device.

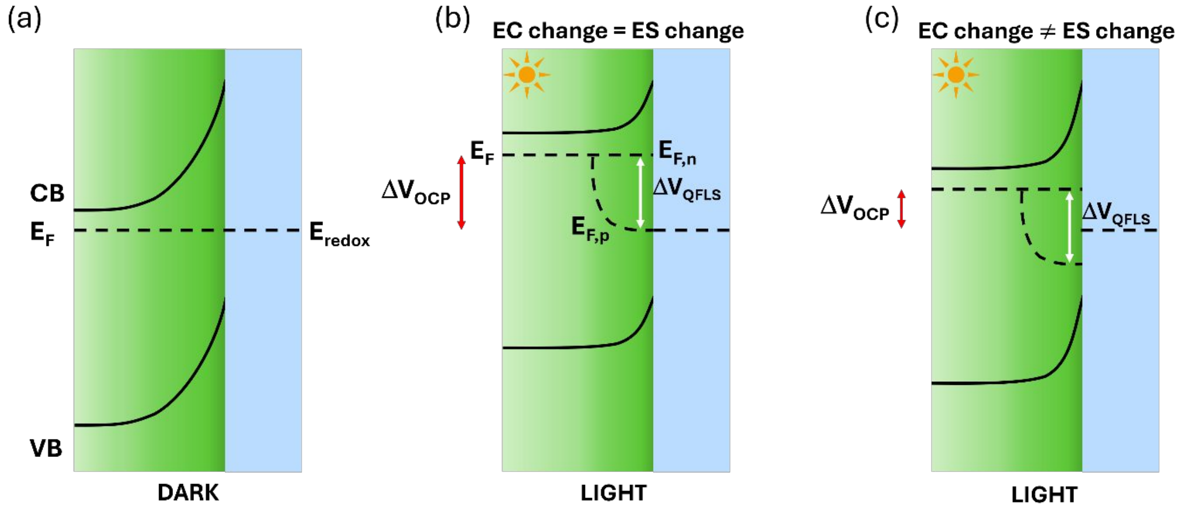

Figure S2. Band diagrams of an n-type semiconductor/electrolyte interface (a) in the dark and (b), (c) under illumination. (b) Shows the case where the change in electrochemical (EC) potential is equivalent to the change in electrostatic (ES) potential. In such a case, the measured change in open circuit potential (ΔV_{OCP}, red arrow) is equivalent to the quasi-Fermi level splitting (ΔV_{QFLS}, white arrow). (c) Shows the case where the change in EC potential is not equivalent to the change in ES potential, so the measured ΔV_{OCP} is not equivalent to the ΔV_{QFLS}. All band diagrams shown are under open circuit conditions.

Figure S3 shows the raw PIA data used to obtain the QFLS in the main paper Figure 3. Over similar light intensity ranges, larger hole signals are measured as the applied anodic bias is increased. When the rate law measured under different applied biases are co-plotted (Figure S8), we observe that at potentials close to the photocurrent onset the system shows only first order behaviour, even at high light intensities. In contrast, at potentials just below the dark current onset, most light intensities fall within the third order region.

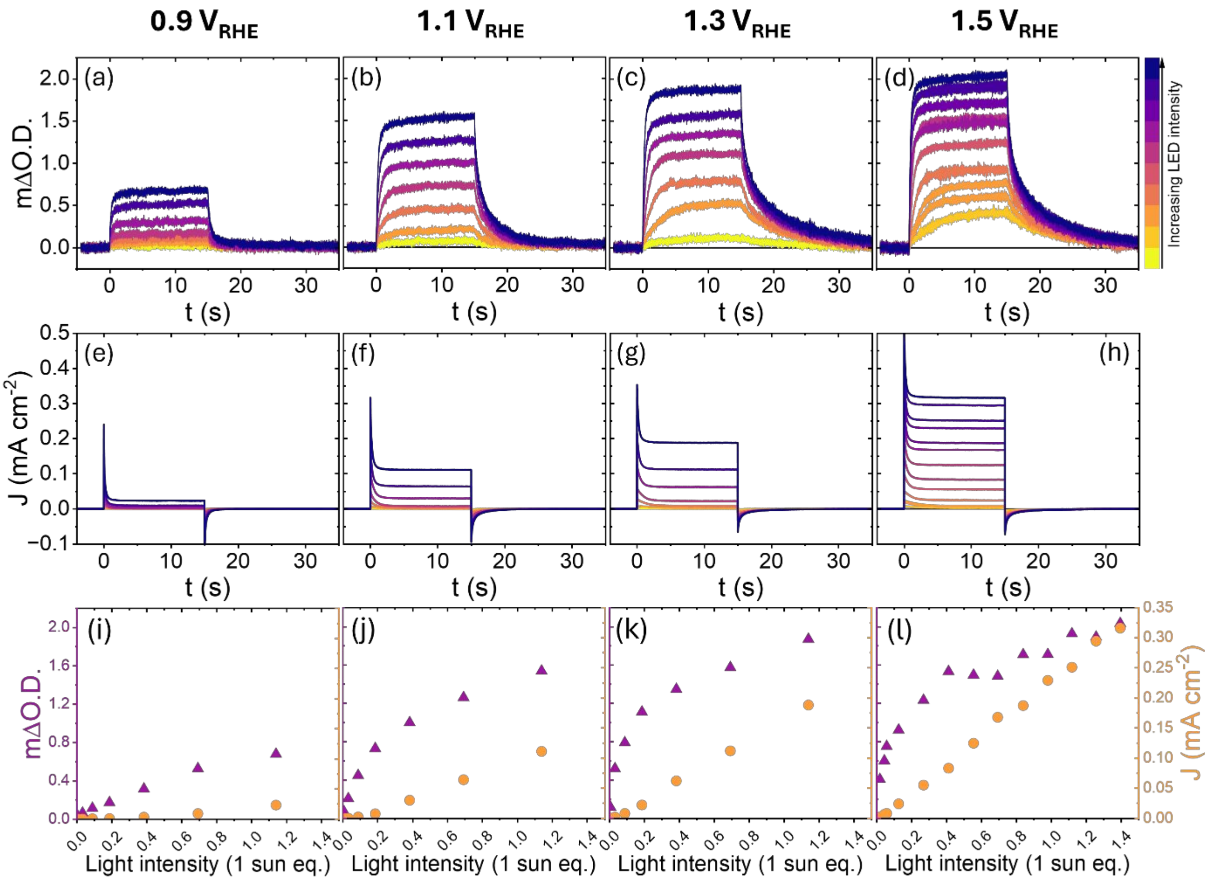

Figure S3. PIA data obtained on the same Fe_2O_3 sample under different applied biases (V_{app}): (a),(e),(i) V_{app} = 0.9 V_{RHE}; (b), (f), (j) V_{app} = 1.1 V_{RHE}; (c), (g), (k) V_{app} = 1.3 V_{RHE}; (d), (h), (l) V_{app} = 1.5 V_{RHE}. (a)-(d) Show optical data (mΔO.D.) under varying LED intensities. (e)-(h) Show current response under varying LED intensities. (i)-(l) Show the steady state mΔO.D. (purple triangles, left y-axis) and steady state current density (orange circles, right y-axis) as a function of light intensity. LED intensities ranged from 0.1 – 15 mW cm^{-2} for 0.9 V_{RHE}, 1.1 V_{RHE} and 1.3 V_{RHE}, and 0.1 – 18 mW cm^{-2} for 1.5 VRHE.

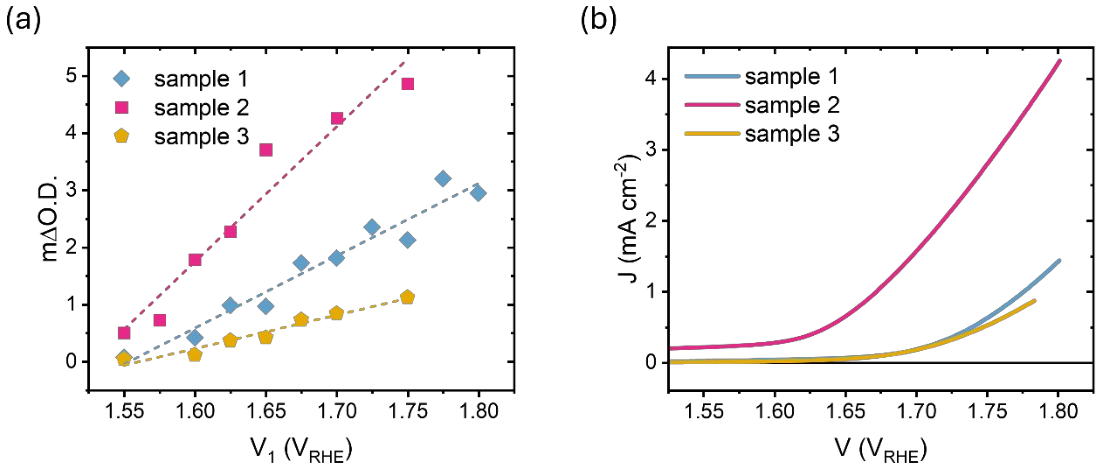

Figure S4. (a) SP-SEC optical data (mΔO.D.) as a function of V_1 for three different hematite samples. V_0 = 1.5 V_{RHE} for all data shown. R^2 = 0.95 (sample 1), 0.94 (sample 2), 0.96 (sample 3). (b) Dark JV curves of the same

samples as in (a), showing the dark current onset. Sample 1 was used to obtain the QFLS data presented in Figure 3 of the main paper.

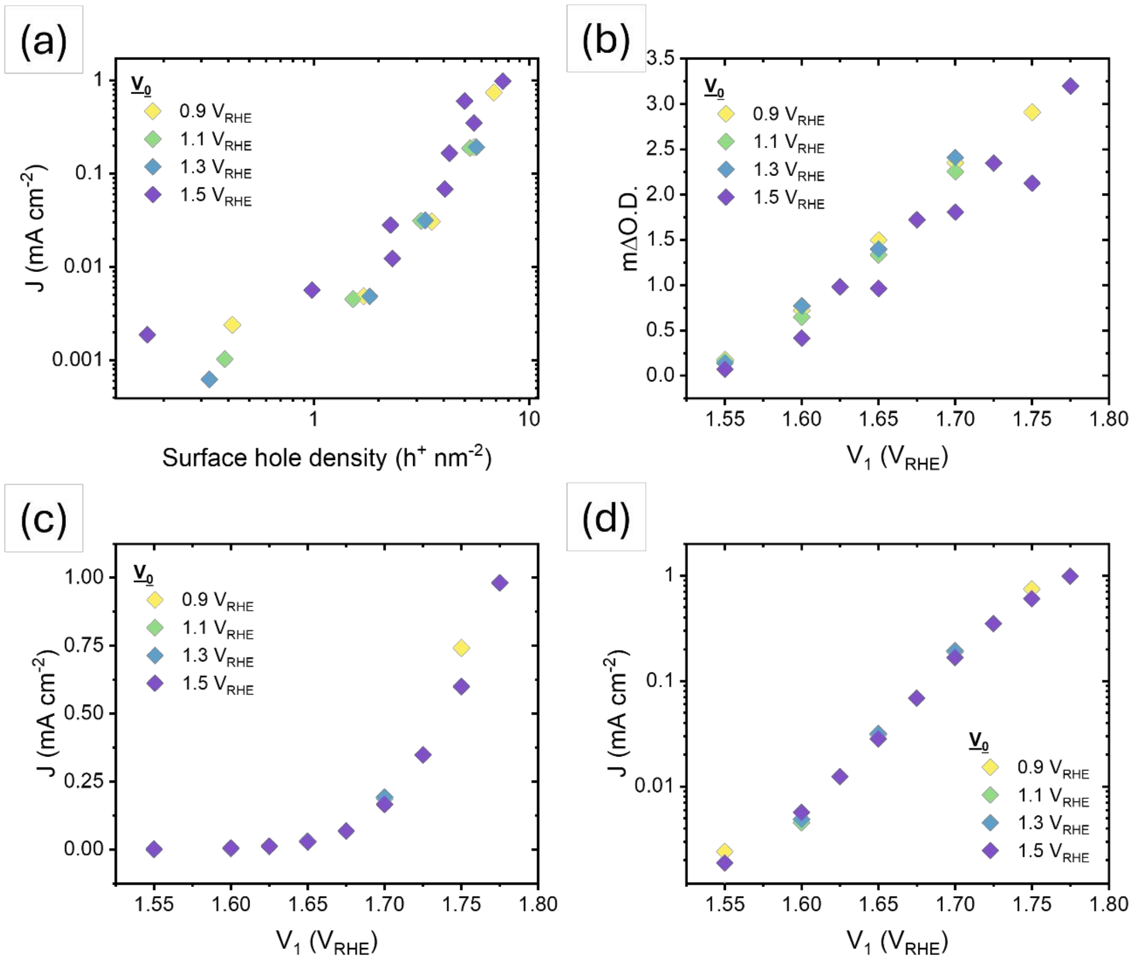

Figure S5. SP-SEC measurements of Fe_2O_3 at different V0 values; 0.9 V_{RHE} (yellow), 1.1 V_{RHE} (green), 1.3 V_{RHE} (blue) and 1.5 V_{RHE} (purple). (a) Rate law plot, (b) optical signal as a function of applied bias, (c) current response (linear) as a function of applied bias and (d) current response (log) as a function of applied bias.

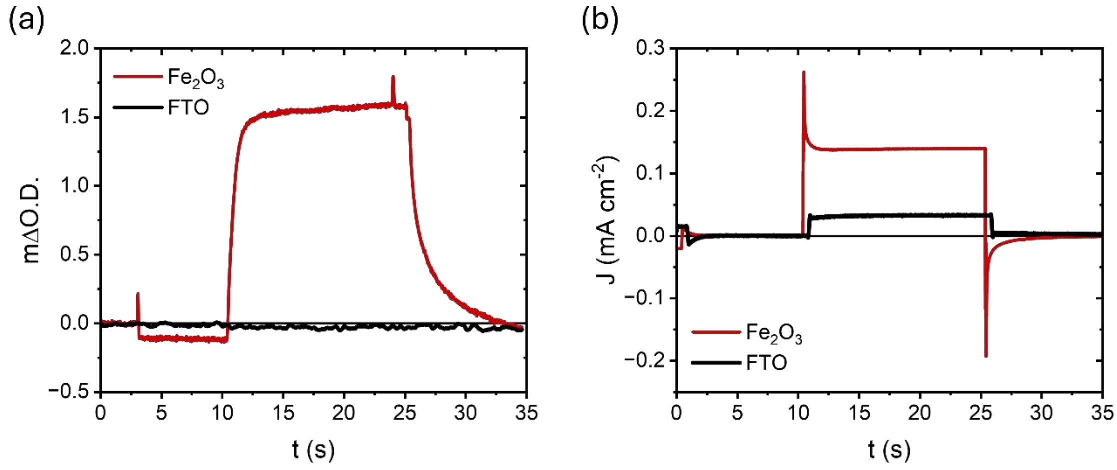

Figure S6. (a) Optical response at 650 nm and (b) current density response of blank FTO (black) and Fe_2O_3 (red) during SPSEC measurements. For both samples, V_0 = 1.5 V_{RHE} and V_1 = 1.7 V_{RHE}. Note that for the conversion of

current to current density for FTO, the area in contact with the electrolyte was approximated to 1 cm^2 (area data not collected for this sample).

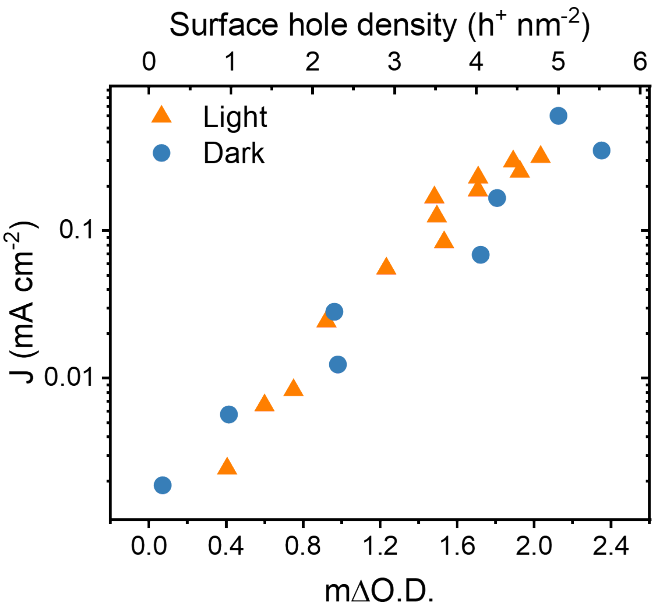

Figure S7. Optical signal (bottom x-axis) and corresponding surface hole density (top x-axis) versus current density for a sample measured in the dark (SP-SEC, blue circles) and light (PIA (front illumination), orange triangles). mΔO.D. converted to surface hole density using a molar extinction coefficient of 640 M^{-1} cm^{-1} from previous work.[4] V_0 (SP-SEC) = V_{app} (PIA) = 1.5 V_{RHE}.

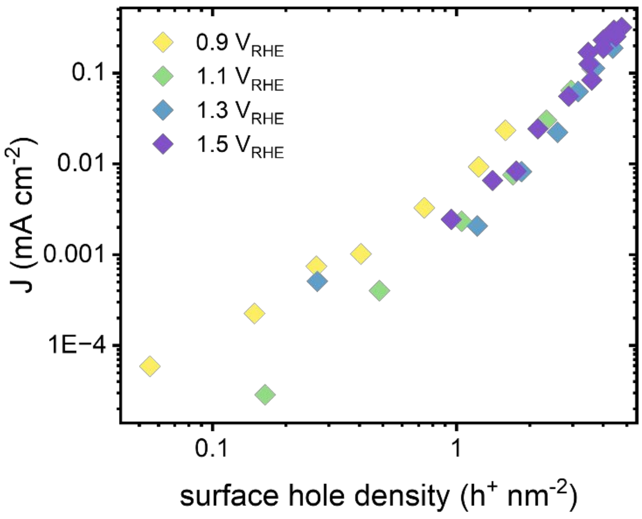

Figure S8. Rate law plot for Fe_2O_3, using PIA measurements under different applied biases: 0.9 V_{RHE} (yellow), 1.1 V_{RHE} (green), 1.3 V_{RHE} (blue) and 1.5 V_{RHE} (purple).

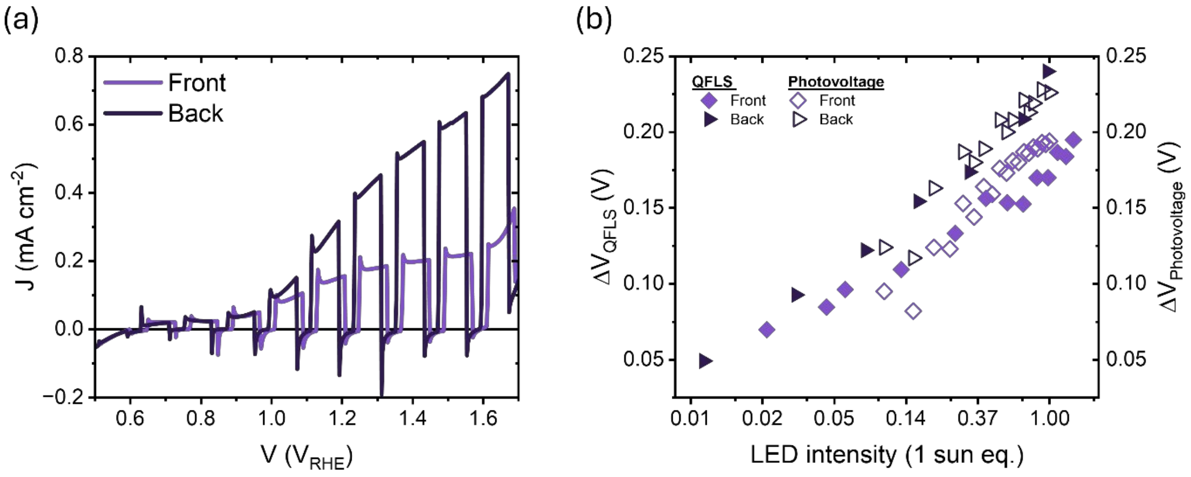

Figure S9. Comparison of Fe_2O_3 performance under front (light purple) and back (dark purple) illumination. (a) Shows the JV curve under chopped 9 mW cm^{-2} (~0.7 sun) illumination and (b) shows the QFLS (solid symbols) and photovoltage (empty symbols) obtained at an applied bias of 1.5 V_{RHE}.

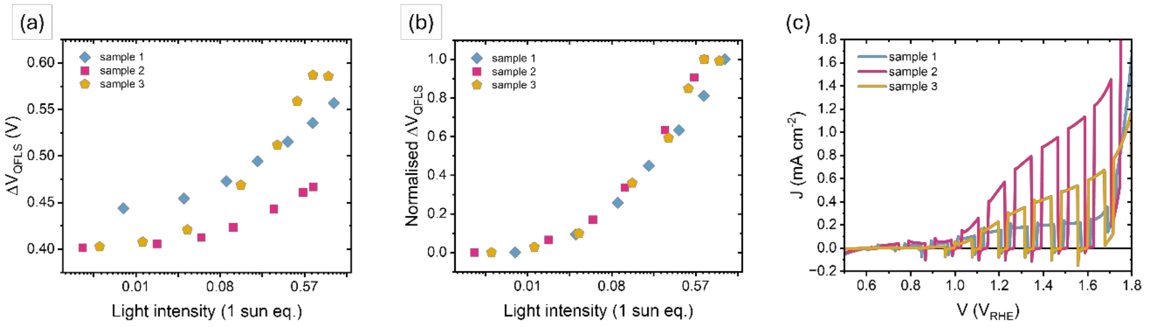

Figure S10. Comparison of QFLS measured from spectroscopy for three different Fe_2O_3 samples, obtained from PIA measurements at Vapp = 1.1 V_{RHE}. (a) Shows the ΔV_{QFLS} as a function of light intensity and (b) shows this same data normalised between 0 and 1. (c) JV curves of the same three samples under chopped 365 nm LED illumination (9 mW cm^{-2} , ~0.7 sun). Sample 1 measured under front illumination, samples 2 and 3 measured under back illumination.

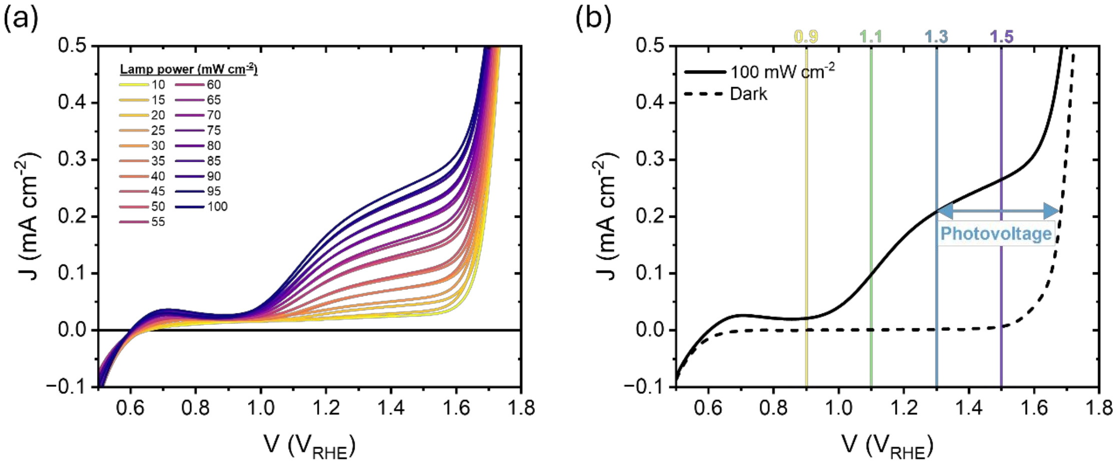

Figure S11. Measuring photovoltage from light and dark JV curves. (a) JV curves of hematite measured at different light intensities. (b) Example light and dark JV curve with vertical lines showing the points along the light JV curve at which the photovoltage was measured. An arrow shows how photovoltage is obtained from these data at 1.3 V_{RHE} in the light.

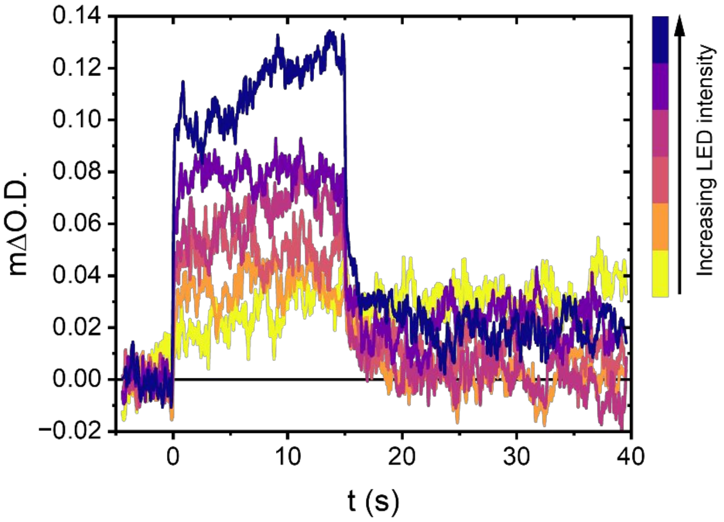

Figure S12. Optical data (probed at 650 nm) obtained during PIA measurements of a hematite sample at open circuit. Front side illumination from a UV LED excitation, varying in intensity from ~0.1-12.6 mW cm^{-2}.

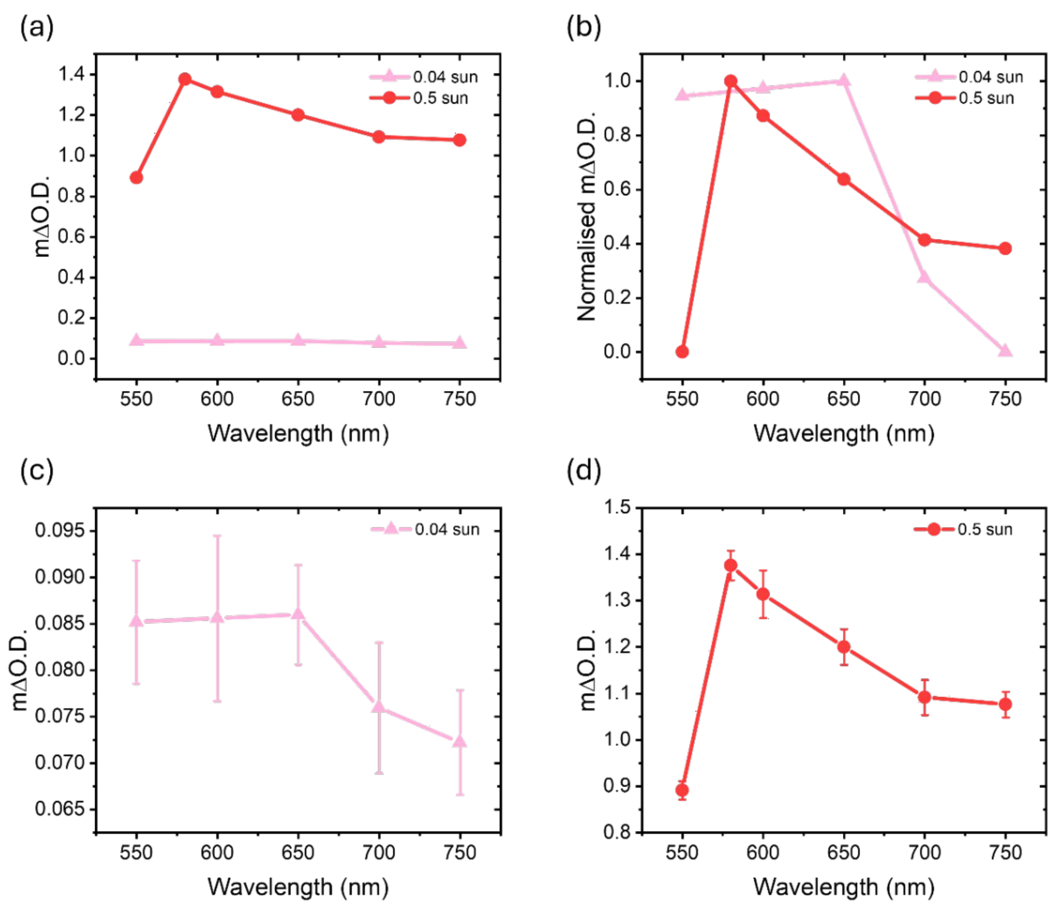

Figure S13. Steady state PIA spectra of Fe_2O_3 obtained under low LED intensity, corresponding with the first order region (0.5 mW cm^{-2} ≈ 0.04 sun, pink triangles) and high LED intensity, corresponding with the third order region (7 mW cm^{-2} ≈ 0.5 sun, red circles). Optical absorption data compared in (a) and normalised between 0 and 1 in (b). (c), (d) show the individual spectra at ~0.04 sun illumination and ~0.5 sun illumination, respectively, with error bars representing one standard deviation. Measurements carried out an applied bias of 1.1 V_{RHE}.

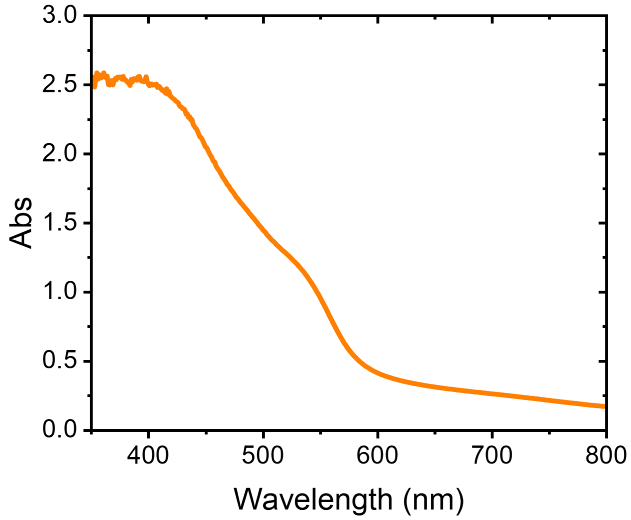

Figure S14. UV-vis spectrum of Fe_2O_3.

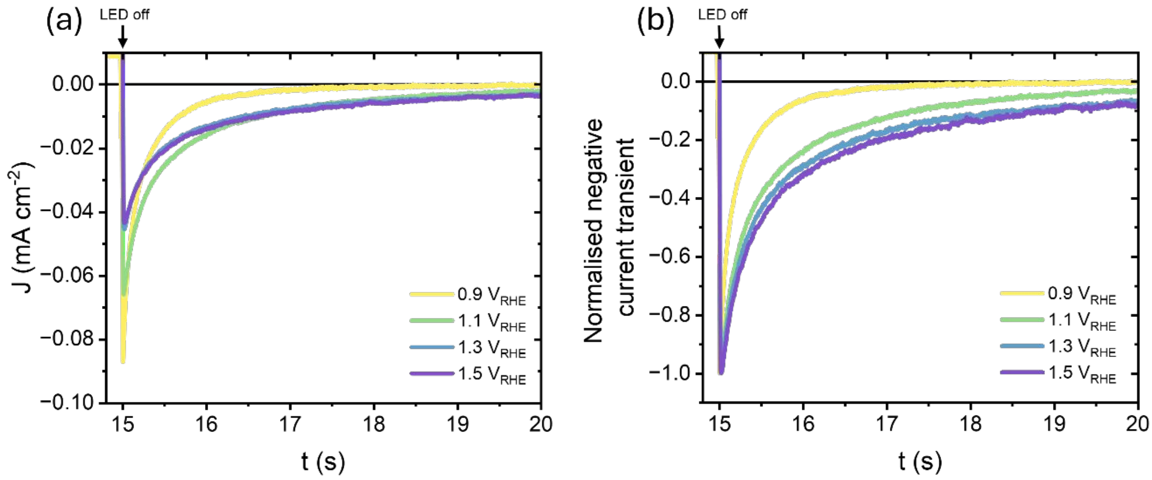

Figure S15. (a) Negative current transients after the LED was switched off during PIA measurements. (b) Shows the same data normalised to their minimum point. Data collected under four different applied biases: 0.9 V_{RHE} (yellow), 1.1 V_{RHE} (green), 1.3 V_{RHE} (blue and 1.5 V_{RHE} (purple). 365 nm LED used at an intensity of ~9 mW cm^{-2} (~0.7 sun) under front illumination.

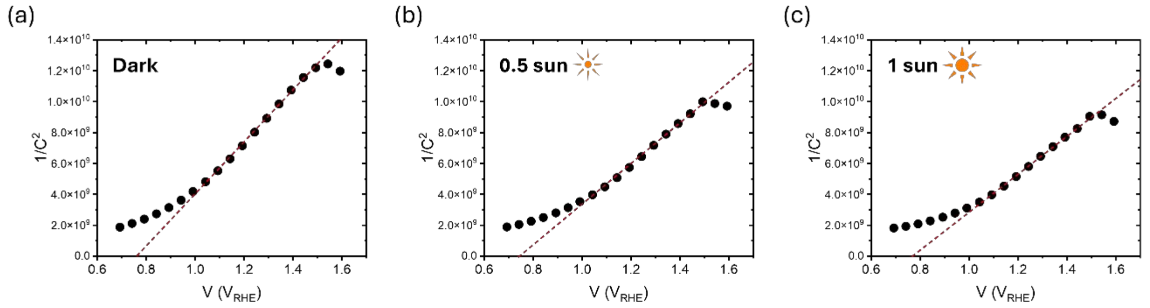

Figure S16. Mott-Schottky measurements obtained at a frequency of 1 kHz (a) in the dark, (b) under 0.5 sun illumination and (c) under 1 sun illumination. 365 nm LED light source.

3. Supplementary Note 1: Comparison of PEC and EC current and hole densities

For the same reaction at the same current density, it has previously been demonstrated that the $E_{F,p}$ of an n-type photoanode can be derived from direct measurements of the electrode potential of a p-type anode in the dark providing three criteria are met: (1) the CB and VB positions are equivalent in the two semiconductors; (2) the reactions at both semiconductors are controlled by the surface hole density; and (3) in the p-type electrode the $E_{F,p}$ is equivalent to the bulk E_F.[5,6]

These principles can be applied to an n-type semiconductor operating under PEC and EC conditions for cases where the same water oxidation kinetics are observed under both conditions, as demonstrated for $BiVO_4$[7] and Fe_2O_3[8]. In both cases, PEC and EC water oxidation are driven by VB holes. Due to the slow kinetics of the multi-redox inner sphere water

oxidation process, large populations of surface holes are accumulated, resulting in an inversion layer at the surface (p-type). We note this behaviour is distinct from that observed with fast outer-sphere redox systems, as investigated in previous work.[5,9,10] As the same material is being investigated under both PEC and EC conditions, the CB and VB positions will be equivalent providing the system is operating under conditions of band edge pinning in both cases. We further note that the overlap of rate laws in the light and dark (Figure 2f and Figure S7) indicates that any IR losses in the system are similar in the light and dark, most likely due to the low current densities, and to the strongly n-type nature of the electrode both reducing bulk resistance and minimising the space charge layer width.[2,11,12] The mechanism for water oxidation at metal oxide photoanodes is proposed to proceed via a series of proton-coupled electron transfer steps, where hole transfer to the semiconductor surface is coupled to proton release and is therefore charge neutral.[13,14] With no net change in surface charge under illumination, the energies of the band edges do not change relative to the redox potential of the electrolyte and the system operates under conditions of band edge pinning. As previous work has shown that the $E_{F,p}$ position is dependent on the surface hole population and that a given current density is correlated with a given surface hole density[5] it follows that the same principles applied to the JV characteristics can be applied to hole populations measured spectroscopically on the same material under PEC and EC conditions.

4. Supplementary Note 2: Ideality of system

The relationship between the photovoltage (V_{photo}) and light intensity (ϕ) can be described by the ideal diode equation:

$$V_{photo} = \left(\frac{nk_BT}{e}\right)\ln(\phi)$$

The ideality factor, n, can be extracted from the slope of V_{photo} versus $\ln(\phi)$, where an ideal system exhibits a slope of ~59 mV per decade (n = 1) at room temperature. The ideality factor is used in the field of solar cells to describe the relationship between V_{OC} and light intensity and is used to provide information on the dominant recombination pathway, with values typically observed to be between 1 and 2. [15–17] In this work, the ideality factor was extracted from the slope of Figure 3a (main paper) and is listed for each applied bias in Table S1. The ideality factors range from 0.4-1.1, increasing with increasing anodic bias. The ideality, when measured this way, is not intrinsic to the material system and depends on the operating conditions.

Table S1. Ideality factor, n, extracted from the slope of Figure 3a (QFLS) at four different applied biases.

V_{app} (V_{RHE})	Slope	Ideality
0.9	0.010	0.4
1.1	0.023	0.9
1.3	0.027	1.0
1.5	0.029	1.1

Another method to assess the ideality of a system is to examine how the surface hole density varies with applied bias as the E_F approaches the VBE. For an ideal band edge, this relationship should follow an exponential trend, as predicted by Ferm-Dirac statistics (Boltzmann function[18]). For the QFLS analysis presented in the main paper, the relationship between the SP-SEC ΔO.D. signal and applied V_1 was approximated to a linear trend within a narrow V_1 range (between the onset of dark current and the VBE). This was reproducible over multiple samples, as shown in Figure S4.

To investigate this relationship further, SP-SEC measurements were obtained at less anodic V_1 values, as shown in Figure 2e in the main paper. These data demonstrate an exponential relationship and have been fit to the equation $m\Delta O.D. = A\exp\left(\frac{V_1 - V_{OCP}}{\alpha}\right)$. For the purposes of this fitting, V_{OCP} was set to 0.8 V_{RHE} (the approximate dark V_{OCP} measured on one of the other samples in this study). According to the Fermi-Dirac model, $\alpha = \frac{n\, k_B\, T}{e}$. At room temperature for an ideal semiconductor (where n = 1), α = 0.026. For the data shown in Figure 2e, α = 0.157, corresponding to an ideality factor of $n \approx 6$. (Note that, from this fitting, A ≈ 0.003.) The Fermi-Dirac distribution assumes an ideal semiconductor in vacuum, so it is perhaps unsurprising that the VBE at a semiconductor/electrolyte interface does not demonstrate ideal behaviour. These observations suggest that the full behaviour of these photoanodes cannot be described by the ideal diode equation and that other physical processes influence photoanode performance, including the filling of surface states.

5. Supplementary Note 3: Control measurements to confirm EC water oxidation occurs at the Fe_2O_3/electrolyte interface

Control SP-SEC measurements were carried out on blank FTO and are compared with equivalent measurements on Fe_2O_3 in Figure S6. For FTO, no change in absorption at 650 nm was observed despite the observed current response (note that the current response is

smaller than that observed on Fe_2O_3 under the same conditions). This shows that, even if pinholes were present in the Fe_2O_3 sample, the FTO would not contribute to the optical signal at 650 nm. Consequently, the SP-SEC measurement (and subsequent calculation of QFLS) is effectively independent of pinholes. Furthermore, as PIA and SP-SEC measurements show that the same hole signal is correlated with the same current density under both PEC and EC conditions (main paper Figure 2f and Figure S7), the FTO must not be contributing the EC current signal which means the derivation of photovoltage from the JV curves of these samples is valid.

6. Supplementary Note 4: Light intensities > 1 sun

This study has focused on the first and third order water oxidation regions. Observations from our own group[19] and others[8] have observed that at higher light intensities (> 1 sun), metal oxides demonstrate higher order behaviour. This behaviour has been attributed to band edge unpinning (Fermi level pinning), which occurs when the proportion of the surface that is oxidised becomes so high that accumulation of further holes can no longer be compensated by the release of protons and the metal oxide demonstrates Butler-Volmer behaviour.[19] Under such conditions, hole accumulation is associated with an increase in net positive charge at the surface and neighbouring charges repel one another. This results in an electrostatic drop within the electrolyte Helmholtz layer (in contrast to the case of band edge pinning where the potential drop occurs only within the SCL of the semiconductor), and the band edge positions shift relative to the redox potential of the electrolyte. This alters the energetics at the surface and changes the band diagram from that shown in Figure 6. An investigation of QFLS at light intensities > 1 sun is beyond the scope of the current study but is the subject of ongoing work.

7. References

(1) Jang, J. W.; Du, C.; Ye, Y.; Lin, Y.; Yao, X.; Thorne, J.; Liu, E.; McMahon, G.; Zhu, J.; Javey, A.; Guo, J.; Wang, D. Enabling Unassisted Solar Water Splitting by Iron Oxide and Silicon. *Nat. Commun.* **2015**, *6*, 7447. https://doi.org/10.1038/ncomms8447.

(2) Liu, T.; Wang, P.; Li, W.; Wang, D. Z.; Lekamge, D. D.; Chen, B.; Houle, F. A.; Waegele, M. M.; Wang, D. Temperature-Dependent Water Oxidation Kinetics: Implications and Insights. *ACS Cent. Sci.* **2025**, *11* (1), 91–97. https://doi.org/10.1021/acscentsci.4c01415.

(3) Liu, T.; Li, W.; Wang, D. Z.; Luo, T.; Fei, M.; Shin, D.; Waegele, M. M.; Wang, D. Low Catalyst Loading Enhances Charge Accumulation for Photoelectrochemical Water Splitting. *Angew. Chem. Int. Ed.* **2023**, *62* (34), e202307909. https://doi.org/10.1002/anie.202307909.

(4) Le Formal, F.; Pastor, E.; Tilley, S. D.; Mesa, C. A.; Pendlebury, S. R.; Grätzel, M.; Durrant, J. R. Rate Law Analysis of Water Oxidation on a Hematite Surface. *J. Am. Chem. Soc.* **2015**, *137* (20), 6629–6637. https://doi.org/10.1021/jacs.5b02576.

(5) Reineke, R.; Memming, R. Comparability of Redox Reactions at N-and p-Type Semiconductor Electrodes. 1. The Quasi-Fermi Level Concept. *J. Phys. Chem* **1992**, *96*, 1310–1317.

(6) Meisner, D.; Memming, R. Analysis of Current-Potential Characteristics at n-and p-Type Semiconductor Electrodes. *Electrochim. Acta* **1992**, *37* (5), 799–809.

(7) Li, B.; Oldham, L. I.; Tian, L.; Zhou, G.; Selim, S.; Steier, L.; Durrant, J. R. Electrochemical versus Photoelectrochemical Water Oxidation Kinetics on Bismuth Vanadate (Photo)Anodes. *J. Am. Chem. Soc.* **2024**, *146* (18), 12324–12328. https://doi.org/10.1021/jacs.4c03178.

(8) Saeed, K. H.; Garcia Osorio, D. A.; Li, C.; Banerji, L.; Gardner, A. M.; Cowan, A. J. Monitoring Interfacial Electric Fields at a Hematite Electrode during Water Oxidation. *Chem. Sci.* **2023**, *14* (12), 3182–3189. https://doi.org/10.1039/d2sc05628c.

(9) Daemi, S.; Kundmann, A.; Becker, K.; Cendula, P.; Osterloh, F. E. Contactless Measurement of the Photovoltage in BiVO4 Photoelectrodes. *Energy Environ. Sci.* **2023**, *16* (10), 4530–4538. https://doi.org/10.1039/d3ee02087h.

(10) Shioiri, Y.; Obata, K.; Kawase, Y.; Higashi, T.; Katayama, M.; Schleuning, M.; van de Krol, R.; Friedrich, D.; Abdi, F. F.; Takanabe, K. Estimating the Quasi-Fermi Level of Holes at the Surface of Semiconductor Photoanodes Using Outer-Sphere Redox Couples. *Nature Communications* **2025**, *16* (1), 3688. https://doi.org/10.1038/s41467-025-58837-9.

(11) Cesar, I.; Sivula, K.; Kay, A.; Zboril, R.; Grätzel, M. Influence of Feature Size, Film Thickness, and Silicon Doping on the Performance of Nanostructured Hematite Photoanodes for Solar Water Splitting. *Journal of Physical Chemistry C* **2009**, *113* (2), 772–782. https://doi.org/10.1021/jp809060p.

(12) Bohn, C. D.; Agrawal, A. K.; Walter, E. C.; Vaudin, M. D.; Herzing, A. A.; Haney, P. M.; Talin, A. A.; Szalai, V. A. Effect of Tin Doping on α-Fe 2O 3 Photoanodes for Water Splitting. *Journal of Physical Chemistry C* **2012**, *116* (29), 15290–15296. https://doi.org/10.1021/jp305221v.

(13) Righi, G.; Plescher, J.; Schmidt, F. P.; Campen, R. K.; Fabris, S.; Knop-Gericke, A.; Schlögl, R.; Jones, T. E.; Teschner, D.; Piccinin, S. On the Origin of Multihole Oxygen Evolution in Haematite Photoanodes. *Nat. Catal.* **2022**, *5* (10), 888–899. https://doi.org/10.1038/s41929-022-00845-9.

(14) Mesa, C. A.; Francàs, L.; Yang, K. R.; Garrido-Barros, P.; Pastor, E.; Ma, Y.; Kafizas, A.; Rosser, T. E.; Mayer, M. T.; Reisner, E.; Grätzel, M.; Batista, V. S.; Durrant, J. R. Multihole Water Oxidation Catalysis on Haematite Photoanodes Revealed by Operando Spectroelectrochemistry and DFT. *Nat. Chem.* **2020**, *12* (1), 82–89. https://doi.org/10.1038/s41557-019-0347-1.

(15) Elumalai, N. K.; Uddin, A. Open Circuit Voltage of Organic Solar Cells: An in-Depth Review. *Energy Environ. Sci.* **2016**, *9* (2), 391–410. https://doi.org/10.1039/c5ee02871j.

(16) Kirchartz, T.; Deledalle, F.; Tuladhar, P. S.; Durrant, J. R.; Nelson, J. On the Differences between Dark and Light Ideality Factor in Polymer:Fullerene Solar Cells. *Journal of Physical Chemistry Letters* **2013**, *4* (14), 2371–2376. https://doi.org/10.1021/jz4012146.

(17) Sze, S. M.; Ng, K. K. P - n Junctions . In *Physics of Semiconductor Devices*; Wiley, 2006; pp 77–133. https://doi.org/10.1002/9780470068328.ch2.

(18) Rajeshwar, K. Fundamentals of Semiconductor Electrochemistry and Photoelectrochemistry. In *Encyclopedia of Electrochemistry*; Bard, A. J., Ed.; Wiley, 2007; Vol. 6.

(19) He, T.; Benetti, D.; Tseng, C.; Moss, B.; Teschner, D.; Jones, T. E.; Kafizas, A.; Grätzel, M.; Piccinin, S.; Durrant, J. R. Observation of Transition from Rate Law to Butler-Volmer Controlled Water Oxidation Kinetics on Hematite Photoanodes. *J. Am. Chem. Soc.* **2026**. https://doi.org/10.1021/jacs.5c18734.
